# The evolving role of genetic tests in reproductive medicine

**DOI:** 10.1186/s12967-019-2019-8

**Published:** 2019-08-14

**Authors:** Federica Cariati, Valeria D’Argenio, Rossella Tomaiuolo

**Affiliations:** 1 KronosDNA srl, Spinoff of Università Federico II, Naples, Italy; 2 Dipartimento di Medicina Molecolare e Biotecnologie Mediche, Università degli Studi di Napoli Federico II, Via Sergio Pansini 5, 80131 Naples, Italy; 3 CEINGE-Biotecnologie Avanzate scarl, Via Gaetano Salvatore 486, 80145 Naples, Italy

**Keywords:** Genetic tests, Reproductive medicine, Male infertility, Female infertility, Assisted reproductive technology

## Abstract

Infertility is considered a major public health issue, and approximately 1 out of 6 people worldwide suffer from infertility during their reproductive lifespans. Thanks to technological advances, genetic tests are becoming increasingly relevant in reproductive medicine. More genetic tests are required to identify the cause of male and/or female infertility, identify carriers of inherited diseases and plan antenatal testing. Furthermore, genetic tests provide direction toward the most appropriate assisted reproductive techniques. Nevertheless, the use of molecular analysis in this field is still fragmented and cumbersome. The aim of this review is to highlight the conditions in which a genetic evaluation (counselling and testing) plays a role in improving the reproductive outcomes of infertile couples. We conducted a review of the literature, and starting from the observation of specific signs and symptoms, we describe the available molecular tests. To conceive a child, both partners' reproductive systems need to function in a precisely choreographed manner. Hence to treat infertility, it is key to assess both partners. Our results highlight the increasing importance of molecular testing in reproductive medicine.

## Introduction

During the last few decades, there have been a series of striking advancements in reproductive and laboratory medicine that have essentially caused these two fields to become inextricably connected. Laboratory medicine now plays a critical role in all stages of the reproductive process, from diagnostic approaches to the choice of the most complex therapy.

**Fig. 1:**
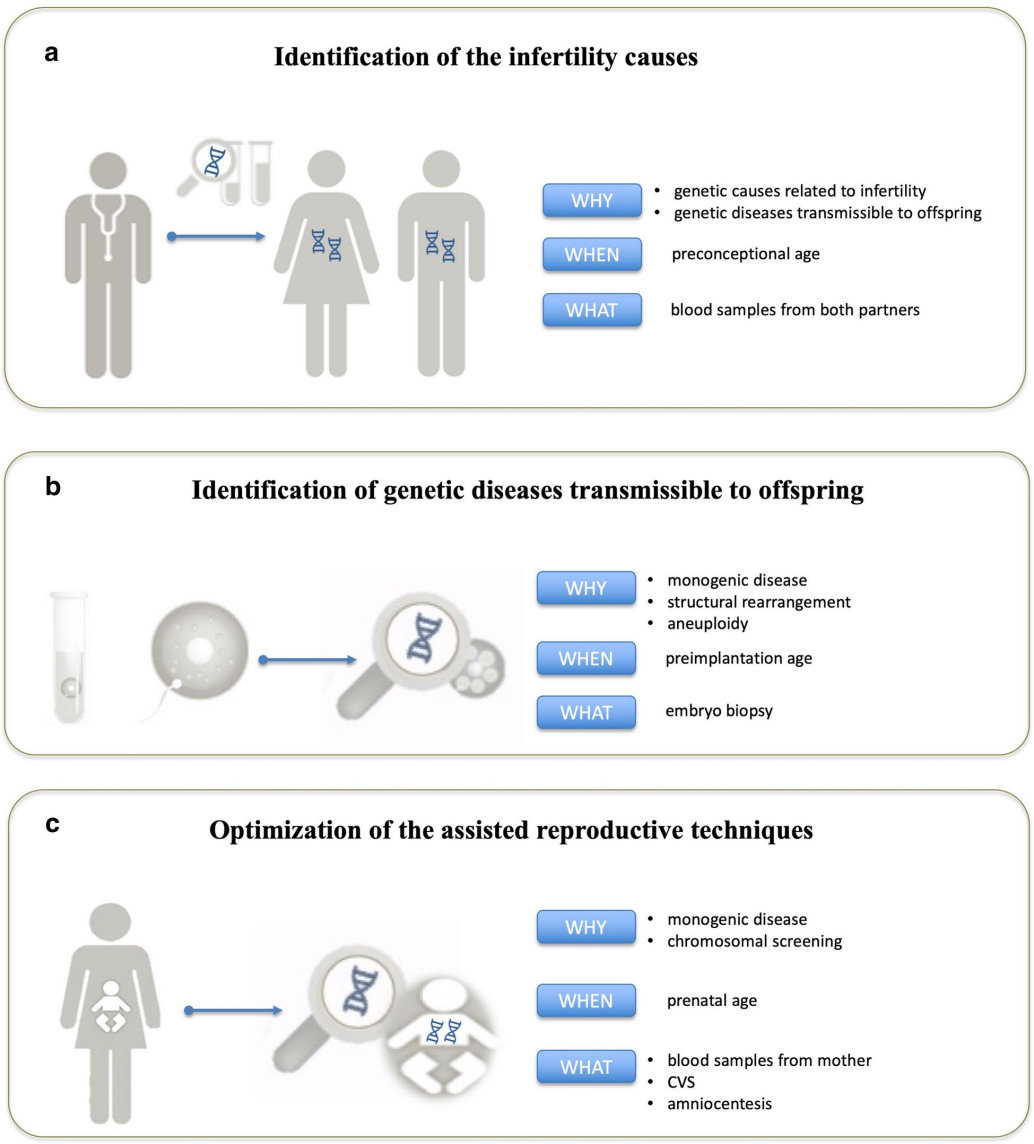
The three main fields of application for which genetic testing is required to improve reproductive medicine: identification of the infertility causes (**a**), identification of genetic diseases transmissible to offspring (**b**), and optimization of the assisted reproductive techniques (**c**)

In particular, genetic tests are carried out for three main purposes in reproductive medicine: the identification of the infertility causes, identification of genetic diseases transmissible to offspring, and optimization of the assisted reproductive technology (ART) (Fig. 1).

The overall fertility rate is decreasing; for example, in the US, 12% of women receive fertility treatment over the course of their lifetimes, so it is important to emphasize the fertility journey of couples [1]. The reproductive systems of both partners function in a combined and precisely coordinated way to conceive a child; for this reason, evaluation of both members of the couple is mandatory.

A medical evaluation is indicated when the couple fails to achieve pregnancy after 12 months of regular, unprotected sexual intercourse [2]. Currently, the diagnostic timeline of infertile couples includes biochemical and instrumental analyses that allow for a diagnosis in 65% of cases; in the remaining 35% of cases, which are undiagnosed, genetic tests are performed. Considering that approximately 15% of genetic disorders are associated with infertility and that similar clinical signs can have genetic and nongenetic causes, it is important that an infertility diagnosis be determined by the combination of an accurate medical history and instrument- and laboratory-based evaluations, including targeted genetic tests [3]. Confirmation of the clinical diagnosis through genetic evaluation (counselling and testing) can lead to more specific and targeted medical management.

In addition, genetic tests are also indicated for the identification of genetic diseases that are transmissible to the offspring: preconception screening allows couples who are planning to become pregnant to know their reproductive risk a priori. Normally, gametes with genetic or chromosomal alterations have reduced reproductive potential. Thanks to ART, many of these difficulties can be overcome, and therefore, genetic tests (carrier screening, preimplantation and prenatal diagnosis) have the crucial impact of monitoring the possible transmission of these genetic alterations to the offspring [4, 5].

Another application field of molecular diagnostics is related to the antenatal diagnosis. To date, the diagnostic options for couples at risk of transmitting a specific inherited disorder to their offspring are preimplantation genetic testing (PGT) and prenatal diagnosis (PND). These two diagnostic procedures share the same purpose but differ in diagnostic time, type of sampling, and laboratory procedures. In addition to the more traditional laboratory investigations, it is now undisputed that molecular biology methods for PGT support the efficacy of ART techniques, contributing significantly to their success (reductions in time, effort and cost) [5, 6].

To optimize the application of genetic tests in clinical practice, in this review, we discuss (1) the genetic conditions related to infertility, including the common and rare ones that are case appropriate; (2) the diagnostic strategies in families at risk of known monogenic disease transmission; and (3) the impact of PGT in the optimization of ART techniques.

## Materials and methods

The literature review was carried out according to PRISMA guidelines. No temporal restrictions were applied. The research was performed using the following keywords: genetic cause of infertility, genetic cause of male infertility, genetic cause of female infertility, mutations and infertility, molecular diagnostics in infertile couples, molecular diagnostics and reproductive medicine, PGT techniques, and PGT and ART. All the papers found were carefully read and evaluated before their inclusion. No unpublished studies were taken into consideration. In addition, the following databases were also consulted to verify gene/phenotype associations: NIH (https://www.nih.gov), OMIM (https://www.omim.org) and OrphaNet (https://www.orpha.net/consor/cgi-bin/index.php).

## Results

### Genetic tests in the identification of the causes of infertility

It has been estimated that every healthy subject is a carrier of 5/8 genetic alterations associated with recessive genetic disorders; therefore, even in the absence of specific symptoms, family planning and reproduction can be risky. Moreover, it has been reported that almost 50% of infertility cases are related to genetic disorders [7, 8]. In the presence or high suspicion of a genetically based reproductive risk, the genetic test provides a more accurate diagnosis of infertility and provides the opportunity to inform the couple about the possible risk of transmission to the offspring. Unfortunately, genetic tests for examining infertility are based on a standard algorithm directed to investigate the most frequent genetic causes without taking into account the patient’s personal or family history. Consequently, the results are quite discouraging: a genetic diagnosis is reached in approximately 4% of all infertile males, and approximately 20% of infertile couples remain undiagnosed. In contrast, an accurate medical and familial history aimed at identifying genetically based syndromes (characterized by typical dimorphisms, associated disabilities and even infertility) could direct patients to specific genetic tests [9]. Starting from this consideration, we examined the genetic disorders related to male and female infertility and subdivided them according to the signs and symptoms observed by the specialist during the first medical examination. Genes associated with specific and rare clinical conditions were not excluded either; they could be useful, in the context of a specific clinical picture, to request an in-depth analysis using a targeted genetic test. Therefore, we provide the main points on the genetic pathology, current test execution modality and management of the patient (Tables 1, 2, 3, 4, 5, 6, 7).

**Table 1:** The chromosome aberrations related to testicular male infertility: from the first observation to the report

Indications for genetic test	Genetic condition	Frequency	Test	Chromosome/genetic alterations	ART	Inheritance	Antenatal test	Differential diagnosis	Refs.
Azoospermia/oligozoospermia; Sertoli cell syndrome type I and type II (presence of some tubules with normal spermatogenesis) and hypospermatogenesis diagnosis by histological evaluation	Microdeletion Y chromosome AZFc	1/2.500; (AZFc 60%, AZFb 15%, AZFb-c 22%, AZFa 3%); 13% of azoospermia cases; 3–7% of oligozoospermia cases	Molecular diagnosis by PCR of STS sequences	Interstitial deletion of AZFc Y region (recombination between palindromes b2 and b4); DAZ, BPY2, PRY2, CDY1	✓: testicular sperm retrieval + ICSI	Y linked	✓	Other causes of azoospermia or oligozoospermia	[10]
Azoospermia; spermatogenesis arrest by histological evaluation	Microdeletion Y chromosome AZFb			Interstitial deletion of AZFb Y region (deletions P5/proximal-P1); RBMY, CDY, HSFY, PRY					
Azoospermia	Microdeletion Y chromosome AZFb-c			Combined deletion AZFb + AZFc (P5/distal-P1 or P4/distal-P1)	✓: donor	NA	NA		
Azoospermia; Sertoli cell syndrome type I diagnosis by histological evaluation (i.e., complete absence of germ cells in seminiferous tubules)	Microdeletion Y chromosome AZFa			Deletion of AZFa Y (recombination between HERV15yq1 and HERV15yq2)					

Database sources: NIH, OMIM and OrphaNet

AZF, azoospermia factor; ✓, yes; ✗, no; NA, not applicable; donor, heterologous fertilization with sperm donor; STS, sequence tagged sites

**Table 2:** The chromosome aberrations related to pretesticular male infertility: from the first observation to the report

Indications for genetic test	Genetic condition	Frequency	Test	Chromosome/genetic alterations	ART	Inheritance	Antenatal test	Differential diagnosis	Refs.
Hypergonadotropic hypogonadism, ↑FSH ↑LH ↓T, azoospermia, oligozoospermia; small testes, infertility, gynecomastia; neurocognitive deficits; metabolic syndrome, type 2 diabetes. Approximately 10% of these subjects have spermatozoa in the ejaculate, and in 30–50% of cases there is intratesticular spermatogenesis	Klinefelter’s syndrome	1/660 newborns; > 5% in severe oligozoospermia; 10% in azoospermia	Karyotype	47,XXY (85–90%) 46,XY/47,XXY mosaicism (6–7%) 46,XX/47,XXY or multiple X aneuploidy (3–8%)	✓ Testicular sperm retrieval + ICSI	De novo mutation	NA	46,XX testicular DSD	[11, 12]
Short stature; gynecomastia, male external genitalia, small testes, cryptorchidism, hypospadias, infertility, ↑FSH ↑LH↓T; azoospermia/oligozoospermia	Nonsyndromic 46,XX Testicular Disorders of Sex Development (De la Chapelle syndrome)	1/20.000; 0,9% in azoospermia; 1–3% normospermia	FISH or CMA	SRY^+^ XX (80–90%)	✗ Testicular sperm retrieval; ✓ heterologous fertilization	AD	✓	Syndromic forms of 46,XX testicular DSD; 45X/46,XY; 47,XXY; 46,XX; sex chromosome mosaicisms; Prenatal exposure of 46,XX fetuses to androgens	[13]
Penoscrotal hypospadias, cryptorchidism, infertility; ↑FSH ↑LH↓T; azoospermia/oligozoospermia				SRY^−^ XX (< 10%)		Unknown	✓		
Short stature; small testes, infertility; ↑FSH ↑LH↓T; azoospermia/oligozoospermia			CMA or molecular diagnostic by PCR	CNV or rearrangements in *SOX9*, *SOX3,* RSPO1 and WNT4 *(rare)*		✓AD for SOX9; AR for RSPO1 or WNT4	✓	46,XX; 46,XY disorders of sex development	[14–18]
Tall stature, delayed development of speech, language or motor skills, autism spectrum disorder, hypotonia, motor tics, clinodactyly, scoliosis, attention deficit hyperactivity disorder; ↑FSH normal or ↓T; from normal to azoospermia; from 0.57 to 77.8% sperm mosaicism, a- or hyper diploidy	Double Y syndrome (Jacobs syndrome)	1/1.000; 0.4% in oligozoospermia	Cytogenetics tests	47,XYY; 46,XY/47,XYY mosaics	✓IVF or ICSI in case of oligospermic patients	Does not have a clear pattern of inheritance	✓	46,XY	[14, 19]
Subfertility or uneventful andrological history; oligozoospermia	Balanced structural chromosome aberrations	5% of infertile men	FISH	t(SRY; X); der(13, 14); der(14, 21); der(14, 15)	✓	NA	✓ PGT	Other causes of oligozoospermia	[20]

Database sources: NIH, OMIM and OrphaNet

✓, yes; ✗, no; NA, not applicable; ICSI, intracytoplasmic sperm injection; IVF, in vitro fertilization; FISH, fluorescence in situ hybridization; PGT: preimplantation genetic testing; CMA, chromosomal microarray analysis

#### Male genetic infertility

Genetic factors have been found in all the etiological categories of male fertility (pre-testicular, testicular and post-testicular): OMIM (Online Mendelian Inheritance in Man) reports more than 200 genetic conditions related to male infertility, ranging from the most common clinical presentations of infertility to the rarest complex syndromes in which signs and symptoms are beyond the reproductive problems [103]. In most cases, infertility is only one of the clinical signs of a complex syndrome; on the contrary, in some genetic conditions, infertility is the main phenotypic feature. Moreover, it is important to monitor these infertile patients over time because a greater morbidity and a lower life expectancy have been described for these infertile patients than for the general population and are most likely caused by the genetic abnormalities involved in male sterility [104].

Today, the presence of alterations in the spermiogram is the first indication for genetic tests, particularly in cases of severe oligospermia (< 5 million/ml) (further parameters are hormonal levels, malformations, recurrent abortions, and family history) [105]. Interestingly, a recent study by Oud et al. highlighted how the number of genes that are definitively linked to the more common phenotypes of oligozoospermia or azoospermia remains limited (50%); the other half are genes involved in teratozoospermia, although the monomorphic forms of teratozoospermia are extremely rare [106].

Genetic disorders related to male infertility include whole chromosomal aberrations (structural or numerical), partial chromosomal aberrations (i.e., microdeletions of the Y chromosome) (listed in Tables 1, 2) and monogenic diseases (listed in Tables 3, 4, 5). In particular, abnormalities in sex chromosomes have a greater impact on spermatogenesis, while mutations affecting autosomes are more related, for example, to hypogonadism, teratospermia or asthenozoospermia and to familial forms of obstructive azoospermia.

**Table 3:** The genetic causes related to pretesticular male infertility: from the first observation to the report

Main indications for genetic test	Hypogonadotropic hypogonadism (CHH)								
Other indications for genetic test	Genetic disorder	Frequency	Genetic test	Genetic alterations	ART	Inheritance	Antenatal test	Differential diagnosis	Refs.
Lack of puberty; micropenis, cryptorchidism; prepubertal testicular volume, absence of secondary sexual features, decreased muscle mass, diminished libido, erectile dysfunction, infertility, low testosterone, estradiol	Kallmann syndrome (olfactogenital syndrome with ano- or hyposmia azoospermia)	Prevalence: 1/30,000; incidence: 1/8000	Molecular diagnosis	ANOS1	✓	X-linked	✓	Syndromes associated with hypogonadotropic hypogonadism	[21, 22]
				CHD7, FGFR1, FGF8, SOX10		AD			
				FEZF1, PROK2, PROKR2		AR			
Obesity, retinitis pigmentosa, postaxial polydactyly, kidney dysfunction, behavioral dysfunction; infertility	Bardet–Biedl syndrome (Laurence–Moon–Biedl syndrome)	1:100,000 North America; 1:160,000 Switzerland; 1:17,500 Newfoundland; 1:13,500 Bedouin, Kuwait	Multigene panel	From BBS1 to BBS19	✓	AR	✓	McKusick–Kaufman syndrome (MKS)	[21, 23–25]
Adrenal insufficiency; cryptorchidism, delayed puberty, infertility	X-linked adrenal hypoplasia congenita	1:12,500	Molecular diagnosis	NR0B1	✓	X-linked recessive pattern	✓	21-hydroxylase deficiency; 11-hydroxylase deficiency	[26]
Diabetes mellitus, hypothyroidism, alopecia totalis, long, triangular face, hypertelorism; dystonias, dysarthria, dysphagia; infertility	Woodhouse–Sakati syndrome (diabetes-hypogonadism-deafness-intellectual disability syndrome)	Unknown	Molecular diagnosis	DCAF17	✓	AR	✓	Perrault syndrome; Deafness and hereditary hearing loss; Gonadotropin-releasing hormone deficiency	[27]
Adult-onset neurodegenerative disorder; hypogonadotropic hypogonadism	Gordon Holmes syndrome (cerebellar ataxia and hypogonadotropic hypogonadism)	Unknown	Molecular diagnosis	RNF216, PNPLA6	✓	AR	✓	Cerebellar ataxia	[28–30]
Cirrhosis, diabetes, cardiomyopathy, arthritis, skin hyperpigmentation; elevated serum transferrin-iron saturation (TS); elevated serum ferritin concentration; infertility	Hemochromatosis (Hemochromatosis Type 1, HFE-Associated Hemochromatosis, HFE-HH)	2–5:1000 northern European ancestry; 1:200–400 non-Hispanic whites, North America	Gene-targeted or molecular diagnosis	HFE (typically p.Cys282Tyr and p.His63Asp can be performed first)	✓	AR	NA	Rarer primary iron overload disorders and secondary iron overload disorders	[31–34]
Azoospermia/oligozoospermia; ↑LH, normal T, hyperandrogenism; feminization of the external genitalia at birth, abnormal secondary sexual development in puberty, and infertility	Androgen insensitivity syndrome (AIS)	2–5:100,000	Screening for AR mutations (> 300)	AR	Donor	X-linked recessive	NA	MRKH syndrome; Hypospadias; MAIS; Undermasculinization of external genitalia and pubertal undervirilization	[35, 36]
Glucocorticoid and mineralocorticoid deficiencies; hypospadias; ambiguous genitalia, infertility	3-β-hydroxysteroid dehydrogenase (HSD) deficiency	Unknown	Molecular diagnosis	HSD3B2	Donor	AR	NA	Ambiguous genitalia	[37]
Deficiencies in GH,TSH, LH, FSH, PrL, and ACTH; hypothyroidism; neonatal hypoglycemia; micropenis without hypospadias, with or without cryptorchidism; short stature and delayed bone maturation; absent/delayed/incomplete secondary sexual development, infertility	PROP1-related combined pituitary hormone deficiency	1:4000 in England and the US	Molecular diagnosis	PROP1	✓	AR	✓	CPHD; isolated growth hormone deficiency; isolated hypogonadotropic hypogonadism	[38, 39]
Ambiguous genitalia or external genitalia that appear female; micropenis and hypospadias; not much facial or body hair; infertility	5-Alpha reductase deficiency (familial incomplete male pseudohermaphroditism, type 2)	Unknown	Molecular diagnosis	SRD5A2	✓	AR	✓	Ambiguous genitalia	[40–42]

Database sources: NIH, OMIM and OrphaNet

✓, yes; ✗, no; NA, not applicable; donor, heterologous fertilization with sperm donor

**Table 4:** The genetic causes related to testicular male infertility: from the first observation to the report

Indications for genetic test	Genetic disorder	Frequency	Genetic test	Chromosome/genetic alterations	ART	Inheritance	Antenatal test	Differential diagnosis	Refs.
Maldescended testes									
Absence of one or both testes from the scrotum; nonobstructive azoospermia; hypogonadotropic hypogonadism	Cryptorchidism	2%; 20% of infertile men; 30/80% of azoospermia	Molecular diagnosis	INSL3; LGR8	✓	AD	✓	Hypogonadotropic hypogonadism; Noonan and Prader–Willi syndrome	[43, 44]
Hypertension, hypokalemic alkalosis; lack of secondary sexual characteristics; testicular feminization	17 alpha(α)-hydroxylase/17,20-lyase deficiency	1 in 1 million	Molecular diagnosis	CYP17A1	Donor	AR	NA	Ambiguous genitalia	[45]
Severe muscular hypotonia, genital hypoplasia, incomplete pubertal development, infertility; cryptorchidism (93%); obesity, mental retardation (late onset)	Prader–Willi syndrome (PWS, Prader–Labhart–Willi syndrome)	1:10,000 to 1:30,000	DNA methylation testing; Cytogenetic/FISH/chromosomal microarray findings: deletion in bands 15q11.2-q13 (70%)	15q11.2 region	Donor	Paternal deletion; maternal uniparental disomy15	✓	Cryptorchidism; Craniopharyngioma	[21, 46–48]
Short stature, facial dysmorphism, congenital heart defects, skeletal defects, webbed neck, mental retardation, bleeding diathesis; early onset	Noonan syndrome-1 (NS1)	1:1000–2500	Gene sequencing starting with PTPN11, followed by SOS1, KRAS and RAF1	PTPN11 (> 50%), SOS1 (10–15%), KRAS (5%), RAF1 (3–17%)	✓	AD	✓	Turner syndrome; cryptorchidism; azoospermia	[49]
Gonadal dysgenesis, ambiguous genitalia, infertility; increased risk of Wilms tumor	Denys–Drash syndrome	Unknown	Molecular diagnosis	WT1	–	AD	✓	Frasier syndrome	[50, 51]
Atrophy of the abdominal muscles, malformations of the urinary tract	Prune–belly syndrome (other names Syndrom of Eagle–Barret; syndrom of Obrinsky)	1/35,000 and 1/50,000 births and 95% of cases occur in males	Molecular diagnosis	CHRM3	NA	–	NA	Megacystis/megaureter or posterior urethral valves	[52, 53]
Osteoporosis; hyperglycemia; ambiguous genitalia	Aromatase deficiency	Unknown	Molecular diagnosis	CYP19A1	✓	AR	✓	Other condition of estrogen deficiency	[54, 55]
Proportionate short stature, delayed closure of fontanelles, prominent forehead, drooping shoulders, abnormal dental development; early onset	Cleidocranial dysplasia	1:1,000,000	Molecular diagnosis	RUNX2 (CBFA1)	✓	AD; de novo pathogenic variant	✓	Pycnodysostosis; mandibuloacral dysplasia; CBFB	[21, 56]
Syndromic without maldescended testes									
Short stature, telangiectatic erythematous skin lesions, high risk for malignancies; early onset; azoospermia or severe oligospermia	Bloom’ s syndrome (Bloom–Torre–Machacek syndrome)	Rare disorder	Molecular diagnosis	BLM	✓	AR	✓	RECQ-mediated genome instability; Ataxia–telangiectasia; Fanconi; anemia; Nijmegen breakage syndrome; Werner syndrome	[21, 57]
Short stature, macrocephaly, distinctive face (small, triangular face with prominent forehead, narrow chin, small jaw), delayed development, speech and language problems, learning disabilities; digestive system abnormalities; micropenis; early onset	Russel–Silver syndrome	Prevalence: unknown; estimated incidence ranges from 1 in 30,000–1 in 100,000 people	Methylation	Methylation involving H19 and IGF2	✓	Sporadic; uniparental disomy	Usually not possible	Intrauterine growth retardation and short stature	[21, 58, 59]
Keratoconus, glaucoma, and myopia as well as from malformations of the brain, skeleton, and kidney; impairment of respiratory functions; infertility (asthenozoospermia and abnormal flagellar morphology)	Primary ciliary dyskinesia (PCD)	Prevalence: 1:16,000; 1:400 in a Volendam population residing in a fishing village of North Holland	Molecular diagnosis	DNAH5 (30%), DNAI1 (10%) and TXNDC3, DNAH11, DNAI2 (rare); 60% gene loci unknown	ICSI	AR	✓	Chronic sinopulmonary disease and bronchiectasis	[21, 60]
Multisystem disorder affecting the skeletal and smooth muscles, the heart, the eyes, and the endocrine and central nervous systems. Mental retardation; infertility	Myotonic dystrophy 1 (Morbus Curschmann–Steinert, Dystrophia myotonica 1, DM1)	1 in 8000	Molecular diagnosis of the CTG repeat expansion in the DMPK gene (> 50 CTG repeats result in DM1)	DMPK	✓	AD	✓	Prader–Willi syndrome, nemaline myopathy, X-linked centronuclear myopathy; DM2; Hereditary distal myopathies; Hereditary myotonia	[21, 61]
Bone marrow failure, hypopigmentation, short stature, physical abnormalities, organ defects (gastrointestinal abnormalities; heart defects; and eye abnormalities, malformed ears and hearing loss); increased risk of certain cancers; and malformations of the reproductive system and infertility	Fanconi anemia	1 in 160,000 (more common among people of Ashkenazi Jewish descent, the Roma population of Spain, and black South Africans)	Molecular diagnosis	FANCA, FANCC and FANCG (90%)	NA	AR; AD:RAD51-related FA; X-linked: FANCB-related FA	✓	Bloom syndrome; ataxia–telangiectasia; NBS; Seckel syndrome; neurofibromatosis 1	[62, 63]
Nonsyndromic infertility									
Abnormal sperm cells (round head and no acrosome) and infertility	Globozoospermia (spermatogenic failure 9)	Rare (1:65,000); common in North Africa: 1:100 cases of male infertility	Molecular diagnosis of DPY19L2, followed by SPATA16	DPY19L2 homozygous deletion, point mutations; SPATA16	✓ ICSI + AOA	AR	✓	Spermatogenic failure	[64, 65]
Abnormal sperm cells (abnormally large and misshapen heads, contains extra chromosomes; multiple flagella, most often four) and infertility	Macrozoospermia (spermatogenic failure 5)	Unknown;1:10,000 males in North Africa	Molecular diagnosis	AURKC mutations (c.144delC, 85%; p.Y248, DR 13%)	Donor	AR	NA	Spermatogenic failure	[66–68]
Primary infertility; multiple morphological abnormalities of sperm flagella (absent, short, coiled, bent, and irregular flagella); asthenozoospermia	Multiple morphological abnormalities of the sperm flagella (spermatogenic failure 18)	Unknown	Molecular diagnosis	DNAH1 mutation (c.8626-1G > A; c.3860 T > G)	✓ ICSI	AR	NA	Ciliary dyskinesia primary	[69]
Genital abnormalities; hypoplasia of Leydig cells; micropenis, hypospadias, bifid scrotum, ambiguous genitalia	Leydig cell hypoplasia (hypergonadotropic hypogonadism due to LHCGR defect)	Unknown	Molecular diagnosis	LHCGR	Donor	AR	✓	Hypergonadotropic hypogonadism	[70, 71]
Asthenozoospermia; absence of any other symptoms	CATSPER-related nonsyndromic male infertility	Unknown	Molecular diagnosis	CATSPER1, GALNTL5	Donor	AR	✓	Male infertility	[69, 72, 73]
Normal general physical examination, absence of clinical findings involving other organ systems; typical female external genitalia, uterus and fallopian tubes normally formed, gonadal dysgenesis; skeletal abnormalities, campomelic dysplasia	Swyer syndrome (46,XY complete gonadal dysgenesis)	1 in 80,000	Molecular diagnosis	*SRY* (15%); MAP3K1 (18%); DHH and NR5A1 (rare)	ART	De novo; rare AD	✓	Ambiguous genitalia and/or sex chromosome-phenotype discordance	[13]
Asthenozoospermia; hearing loss	Deafness-infertility syndrome (DIS)	Unknown	CMA/array-CGH	Homozygous deletion at 15q15.3 including CATSPER2, STRC	Donor	AR	✓	DFNB16	[74, 75]
Nonobstructive azoospermia									
Small testes and infertility, with severe oligozoospermia or nonobstructive azoospermia due to maturation arrest at the primary spermatocyte stage	Meiotic arrest at primary spermatocyte stage (spermatogenic failure 25)	Unknown	Molecular diagnosis	TEX11	Donor	X-linked	NA	Spermatogenic failure	[76–78]
Nonobstructive azoospermia, infertility, testicular biopsy showing absence of spermatogenic cells and a Sertoli cell-only pattern	Spermatogenic failure 32	Unknown	Molecular diagnosis	SOHLH1	Donor	AD	NA	Spermatogenic failure	[79]
Azoospermia; testicular histology showing arrest of spermatogenesis at the pachytene stage of primary spermatocytes	Spermatogenic failure 4 (SPGF4)	1%	Molecular diagnosis	SYCP3 (COR1 RPRGL4 SCP3 SPGF4)	Donor	AD	NA	Spermatogenic failure	[80]
Azoospermia or oligozoospermia	Spermatogenic failure, Y-linked 2	Unknown	Molecular diagnosis	RBMY1A1, DAZ1–4	Donor	Y-linked	NA	Spermatogenic failure	[81, 82]

Database sources: NIH, OMIM and OrphaNet

✓, yes; ✗, no; NA, not applicable; donor, heterologous fertilization with sperm donor; AOA, assisted ovarian activation, CMA, chromosomal microarray analysis

**Table 5:** The genetic causes related to posttesticular male infertility: from the first observation to the report

Main indications for genetic test	Obstructive azoospermia or severe oligospermia				ART	Inheritance	Antenatal test	Differential diagnosis	Refs.
	Genetic disorder	Frequency	Genetic test	Genetic alteration					
Abnormalities of seminal vesicles or absence of vas deferens; normal testicular development and function; normal spermatogenesis; a low volume of ejaculated semen with a specific profile (volume < 1.5 ml, ph < 7.0, elevated citric acid concentration, elevated acid phosphatase concentration, low fructose concentration, and failure to coagulate)	Congenital bilateral absence of the vas deferens (CBAVD)	25%; 1–2% in infertility	Screening for CFTR mutations	Two CFTR pathogenic variants identified (46%); one CFTR pathogenic variant identified (79%)	✓ ICSI	AR	✓	Young syndrome; Hereditary urogenital dysplasia	[83–85]
Multisystem disease affecting epithelia of the respiratory tract, exocrine pancreas, intestine, hepatobiliary system, and exocrine sweat glands; obstructive azoospermia and male infertility	Cystic fibrosis	1:3200; CF occurs with lower frequency in other ethnic and racial populations (1:15,000 African Americans, and 1:31,000 Asian Americans)	Screening for CFTR mutations	Two CFTR pathogenic variants identified	✓ ICSI	AR	✓	Asthma; congenital airway anomalies; primary ciliary dyskinesia; Shwachman–Diamond syndrome; Bronchiectasis with or without elevated sweat chloride; Isolated hyperchlorhidrosis; Congenital bilateral absence of the vas deferens (CBAVD)	[84, 86]

Database sources: NIH, OMIM and OrphaNet

✓, yes; ✗, no; NA, not applicable; ICSI, intracytoplasmic sperm injection

Currently, the main genetic tests routinely used for the diagnosis of male infertility are the karyotype, the study of chromosome Y microdeletions, and the analysis of the *CFTR* gene. Since it has been reported that several mutated are related to male infertility, it is not surprising that in ~ 40% of all cases of male infertility, the underlying genetic pathogenesis remains unknown [107, 108]. It must also be considered that the role of de novo mutations should be further investigated, especially in light of what happens for Klinefelter syndrome and AZF deletions that occur almost exclusively de novo [106]. Therefore, to improve and personalize the entire diagnostic–therapeutic pathway of male infertility, targeted genetic tests should be performed in the presence of specific clinical pictures, always after appropriate genetic counselling: (1) for diagnostic purposes, (2) during clinical decision-making to establish the most appropriate ART strategy (for example, in the presence of deletions of the AZFa and AZFb regions, the possibility of sperm recovery using testicular biopsy is extremely low), and (3) for prognostic purposes (to establish the risk of transmitting the pathology and plan a prenatal or preimplantation diagnostic procedures).

*Whole chromosomal aberrations* The prevalence of chromosomal alterations varies from 1.05 to 17% (this gap depends on the characteristics of the studied group) but is 0.84% in newborns [109]. Structural chromosomal rearrangements are more common with respect to numerical abnormalities; this does not apply to sex chromosomes whose abnormalities, accounting for approximately 4.2% of all whole chromosomal aberrations, are represented by sex chromosome aneuploidies in 84% of cases and by structural rearrangements of chromosome Y in the remaining 16% of cases. Klinefelter syndrome (karyotype 47, XXY) is the most frequent type of sex chromosome aneuploidy detected in infertile men [11, 12]; the second most frequent gonosomal abnormality is Double Y syndrome or Jacobs syndrome, characterized by the presence of Y chromosome disomy [14, 110]. In addition to reduced reproductive potential, carriers of chromosomal abnormalities have an increased risk of abortion or generate a child with an abnormal karyotype. For this reason, Table 1 shows the main chromosomal aberrations that could interfere with healthy reproduction, the relative information on the phenotypic aspect, the laboratory tests to highlight them and the indications for antenatal genetic testing.

*Partial chromosomal aberrations* Microdeletions in the long arm of the Y chromosome (Yq), named the AZF (Azoospermia Factor) region, have been found in 8–12% of azoospermic men and 3–7% of oligozoospermic men [106], resulting in the most common molecular genetic cause of male infertility [110]. The AZF region includes three groups of genes (AZFa, AZFb and AZFc) that are most responsible for spermatogenesis, so partial or complete deletions in this area may impair reproductive capacity. Indications for AZF deletion screening are based on sperm count (< 5 × 10^6^ spermatozoa/ml) associated with primary testiculopathy, and ICSI is required to overcome infertility [111].

Male offspring will carry the same father’s Yq microdeletions or even a worse one; therefore, genetic counselling is mandatory [112]. Parents should be aware of the risk of having a child affected by Turner’s syndrome (45, X0) or other phenotypic anomalies associated with sex chromosome mosaicism [113].

The rearrangement of the AZFc zone is responsible for 60% of all Yq microdeletions [114]. The AZFc region (3.5 Mb) contains several copies of five repeats (b1, b2, b3, b4, and gr), whose similarity and large size predispose an individual to a relatively high incidence of de novo deletions via homologous recombination [115]. The most common is the loss of the whole b2/b4 region, which includes the DAZ family (Deleted in Azoospermia) and leads to spermatogenesis deterioration [115, 116]. More details about AZF are reported in Table 1.

*Single gene mutations* This section will focus on the noteworthy single gene disorders that have clinical relevance for male infertility (Tables 3, 4, 5). Although thousands of genes are involved in male infertility, today, only a handful of genetic diseases are routinely investigated (e.g., cystic fibrosis) [117, 118]. As shown by several studies, the approaches to identify a single causative gene are not useful considering that more than 2300 genes are expressed in the testis alone and that hundreds of them influence reproductive functions and can contribute to male infertility. Even if nearly 50% of infertility cases are due to single or multiple genetic defects, the genetic causes remain unexplained for 20% of patients [3]. Furthermore, the increasingly widespread use of tools, such as NGS (next-generation sequencing), for both diagnostic and research purposes will allow us to rapidly expand our knowledge of this field [4].

Starting from the clinical and laboratory evaluation, as shown in Tables 3, 4, 5, the main genetic conditions that could interfere with healthy reproduction are reported with the aim of improving the targeted genetic test in the presence of specific clinical pictures.

#### Female genetic infertility

In contrast to male infertility, little is known about the genetic bases of female infertility. Accordingly, fewer specific tests are routinely recommended to infertile females to investigate the presence of chromosomal disorders or single-gene defects related to their clinical phenotypes. Indeed, isolated infertility due to genetic causes is rare; more commonly, syndromic diseases contribute to female infertility. To date, genetic tests are mainly used for patients with POI, limited to chromosomal aberrations and FMR1 premutations. We therefore focused on the description of these two conditions; however, as shown in Tables 6, 7, more details have been reported concerning the main chromosomal and genetic alterations that could interfere with healthy reproduction; for each of them, the main phenotypic presentations and the laboratory tests that are available in the pre- and postnatal periods are reported.

**Table 6:** The genetic causes related to ovarian female infertility: from the first observation to the report

	Indications for genetic test	Genetic disorder	Frequency	Genetic test	Chromosome/genetic alterations	ART	Inheritance	Antenatal test	Differential diagnosis	Refs.
POI	Short stature, skeletal abnormalities, kidney problems, webbed neck, lymphedema; ovarian hypofunction or premature ovarian failure, infertility	Turner (45,X) (other names monosomy X, TS)	1 in 2500	Karyotype	Monosomy X: 45,X0	✓-donor	Not inherited	NA	POF	[87]
	Asymptomatic (only 10% of individuals with trisomy X are actually diagnosed); tall stature, epicanthal folds, hypotonia and clinodactyly; renal and genitourinary abnormalities; psychological problems	Trisomy X	1/1000	Karyotype	47XXX or mosaic	✓	NA	✓		
	Irregular menstrual cycles, early menopause, premature ovarian failure, infertility	Fragile X-associated primary ovarian insufficiency (premature ovarian failure 1)	1 in 200 (4/6% of all cases of POI)	Molecular diagnosis of premutations in the FMR1 gene on chromosome Xq27.3 (CGG segment is repeated 55 to 200 times)	*FMR1* gene	✓-donor	X-linked	✓	POF	[88]
	Hypogonadotropic hypogonadism; hypotonia, poor feeding, vomiting, weight loss, jaundice; impaired growth, cognitive deficit and cataracts	Galactosemia (galactose-1-phosphate uridyltransferase deficiency)	prevalence unknown; incidence 1/40,000–60,000	Molecular diagnosis	*GALT, GALK1*, and *GALE* genes (9p13, 17q24, 1p36)	✓	AR	✓	POF	
	Chronic mucocutaneous candidiasis, hypoparathyroidism and autoimmune adrenal failure; early onset	Autoimmune polyglandular syndrome (types 1)	Prevalence: 1–9 in 1,000,000; 1/25,000 in Finland	Molecular diagnosis	*AIRE* gene (21q22.3)	✓	AR	✓	IPEX syndrome; autoimmune polyendocrinopathy type 2	
	Hypertension, hypokalemia; abnormal sexual development, amenorrhea, infertility	17α-hydroxylase deficiency	1 in 1 million	Molecular diagnosis	CYP17A1 gene	Donor	AR	NA	Severe congenital adrenal hyperplasias	[45]
	Mineralization of bones and osteoporosis; hyperglycemia; ambiguous genitalia, ovarian cysts early in childhood, anovulation; hirsutism	Aromatase deficiency	unknown	Molecular diagnosis	*CYP19A1* gene	Donor	AR	NA	PCOS	[62, 63]
	Ophthalmic disorder associated with premature ovarian failure; early onset	Blepharophimosis, ptosis, epicanthus inversus syndrome type I (BPES, type I)	Prevalence: 1–9/100 000	Molecular diagnosis	*FOXL2* gene	✓	AD or de novo	✓	PCOS	[89]
	Pre- and postnatal growth retardation, facial sun-sensitive telangiectatic erythema, increased susceptibility to infections, and predisposition to cancer	Bloom syndrome	Unknown; 1/48,000 among people of Ashkenazi Jewish descent	Cytogenetic or molecular diagnosis	15q26.1; *BLM* gene	✓	AR	✓	Silver–Russell syndrome, Rothmund–Thomson syndrome, ataxia–telangiectasia, Cockayne syndrome, and Nijmegen breakage syndrome	
Ovulation disorders (not POI)	Hypergonadotropic amenorrhea; lack of puberty; absence of secondary sexual features, decreased muscle mass, diminished libido, infertility	Kallmann	prevalence: 1/30,000; incidence: 1/8,000	Molecular diagnosis	Type 1: ANOS1 Type 2 and 6: CHD7, FGFR1, FGF8 and SOX10 Type 3: FEZF1, PROK2, PROKR2	✓	X-linked AD AR	✓	Syndromes associated with hypogonadotropic hypogonadism	
	Diabetes mellitus, hypothyroidism, alopecia totalis, long, triangular face, hypertelorism; dystonias, dysarthria, dysphagia	Woodhouse–Sakati syndrome	Unknown	Molecular diagnosis	DCAF17 gene	Donor	AR	NA	Diabetes; hypogonadism; deafness-intellectual disability	[27]
	Hearing loss; intellectual disability, ataxia, peripheral neuropathy; ovarian dysgenesis, primary amenorrhea, primary ovarian insufficiency, normal external genitalia, infertility	Perrault syndrome	Rare	Molecular diagnosis	TWNK; CLPP; HARS; LARS2; HSD17B4	Donor	AR	NA	Gonadal dysgenesis; sensorineural deafness	[90]
		Gonadal dysgenesis, XX type, with deafness								
		Ovarian dysgenesis with sensorineural deafness								
	Primary amenorrhea, infertility, polycystic ovarian syndrome, hirsutism, ambiguous genitalia	Cytochrome P450 oxidoreductase deficiency	Unknown	Molecular diagnosis	*POR* *gene*	Donor	AR	NA	PCOS	[91]
	Skeletal abnormalities, craniosynostosis, a flattened mid-face, a prominent forehead, and low-set ears; arachnodactyly, choanal atresia; primary amenorrhea, infertility, polycystic ovarian syndrome, hirsutism, ambiguous genitalia	Antley–Bixler syndrome	Unknown	Molecular diagnosis	FGFR2 gene	Donor	AR	NA	PCOS	[92]
	Obesity, hirsutism, and amenorrhea are clinical correlates of enlarged polycystic ovaries	Polycystic ovary syndrome (PCOS)	6 to 10% of women worldwide	Molecular diagnosis	AOPEP; AR; DENND1A; ERBB4; FSHB; FSHR; FTO; GATA4; HMGA2; INSR; KRR1; LHCGR; RAB5B; RAD50; SUMO1P1; SUOX; THADA; TOX3; YAP1	✓	Does not have a clear pattern of inheritance	NA	Amenorrhea	[93, 94]
		Polycystic ovary syndrome 1 (STEIN-LEVENTHAL SYNDROME HYPERANDROGENEMIA)		Molecular diagnosis	PCOS1	✓	AD	NA	Amenorrhea; HYPERANDROGENEMIA	[93, 94]
	Hydropic placental villi, trophoblastic hyperplasia, and poor fetal development	Recurrent hydatidiform mole-type 1 (familial recurrent hydatidiform mole, FRHM)	1:250 in eastern Asia	Molecular diagnosis	NLRP7 gene (55%); KHDC3L gene (5%)	✓	AR	✓	Hydatidiform mole	[95, 96]
	Abnormally developed embryo and placenta that result in the formation of hydatidiform moles	Hydatidiform mole	1:1500 in USA	Molecular diagnosis	C11 or F80, MEI1, REC114	✓	AR	✓	FRHM	[95, 96]
	Normal general physical examination, absence of clinical findings involving other organ systems; typical female external genitalia, normally formed uterus and fallopian tubes, gonadal dysgenesis; skeletal abnormalities, campomelic dysplasia	Swyer syndrome (46,XY complete gonadal dysgenesis)	1 in 80,000	Molecular diagnosis	*SRY* (15%); MAP3K1 (18%); DHH and NR5A1 (rare)	ART	De novo; rare AD	✓	Ambiguous genitalia and/or sex chromosome-phenotype discordance	[69]

Database sources: NIH, OMIM and OrphaNet

✓, yes; ✗, no; NA, not applicable; POF, premature ovarian failure; PCOS, polycystic ovarian syndrome

**Table 7:** The genetic causes related to postovarian female infertility: from the first observation to the report

Indications for genetic test	Genetic disorder	Frequency	Genetic test	Genetic alterations	ART	Inheritance	Antenatal test	Differential diagnosis	Refs.
Underdeveloped or absent uterus and abnormalities of other reproductive organs; normal female external genitalia, breasts; hyperandrogenism; facial hirsutism; primary amenorrhea; infertility	Müllerian aplasia and hyperandrogenism (other names: Biason–Lauber syndrome, WNT4 deficiency)	Rare	Molecular diagnosis	WNT4 gene	NA	AD or de novo	✓	Abnormalities of the reproductive system	[97–99]
Vagina and uterus to be underdeveloped or absent, although external genitalia are normal, primary amenorrhea	Mayer–Rokitansky–Küster–Hauser (MRKH) syndrome (type 1)	1 in 4500	Molecular diagnosis	ESR1, OXTR, WNT9B	NA	AD	✓	Abnormalities of the reproductive system	[100–102]
Underdeveloped or absent vagina and uterus, although external genitalia are normal; primary amenorrhea; unilateral renal agenesis; skeletal abnormalities; hearing loss or heart defects	Mayer–Rokitansky–Küster–Hauser (MRKH) syndrome (type 2)								
Bone marrow failure, hypopigmentation, short stature, physical abnormalities, organ defects (gastrointestinal abnormalities; heart defects; eye abnormalities, malformed ears and hearing loss), and an increased risk of certain cancers; abnormal genitalia or malformations of the reproductive system and infertility	Fanconi anemia (Fanconi pancytopenia Fanconi panmyelopathy)	1 in 160,000 (more common among people of Ashkenazi Jewish descent, the Roma population of Spain, and black South Africans)	Molecular diagnosis	FANCA, FANCC and FANCG (90%)	NA	AR; AD (RAD51-related FA); X-linked (FANCB-related FA).	✓	Bloom syndrome; ataxia–telangiectasia, Nijmegen breakage syndrome (NBS); Seckel syndrome; neurofibromatosis 1; POI	[62, 63]

Database sources: NIH, OMIM and OrphaNet

✓, yes; ✗, no; NA, not applicable; POI, primary ovarian insufficiency

*Whole chromosomal aberrations* Considering that chromosomal disorders significantly impact fertility and the miscarriage risk, karyotype analysis is always advisable [119]. The most clinically important structural disorders in infertile females are translocations, both reciprocal (exchange of two terminal segments from different chromosomes) or Robertsonian (centric fusion of two acrocentric chromosomes) responsible for blocks of meiosis and structural alterations of the X chromosome. Patients with reciprocal translocations are at a significantly increased risk of infertility, including hypogonadotropic hypogonadism with primary or secondary amenorrhea or oligomenorrhea. The balanced rearrangements do not create health problems for their carriers because they cause neither loss nor duplication of genetic information, but they can give rise to gametes in which the genetic information is unbalanced and can thus become a cause of infertility or multiple miscarriage. Some abnormalities, such as the XXX karyotype, could not be clearly associated with infertility.

Women with a normal karyotype produce a variable percentage of oocytes with chromosomal abnormalities due to errors occurring during *crossing*-*over* and/or meiotic nondisjunction [120, 121]. The three main classes of abnormalities are 45X, trisomy and polyploidy. It is well known that these events increase with maternal age [122]. It is possible to analyze gametes or embryos while undergoing ART thanks to PGT. The efficacy of the technique is increased after screening for aneuploid embryos and transferring only euploid embryos [123, 124].

*Fragile X syndrome* Fragile X syndrome is an autosomal dominant genetic disorder caused by the presence of over 200 repetitions of the CGG triplet sequence in the *FMR1* (Fragile X Mental Retardation 1) gene or by a deletion affecting the *FMR2* (Fragile X Mental Retardation 2) gene. Carriers of the female *FMR1* premutation (when the number of CGG repeats falls between 55 and 200) or *FMR2* microdeletion show menstrual dysfunction, diminished ovarian reserve, and premature ovarian failure [125, 126].

In addition to the family history, in the case of women with these clinical manifestations, the possibility of a molecular test should be considered. The most common genetic contributors to POI are X-chromosome-linked defects. In rare cases, the cause is an alteration in an autosomal chromosome [88]. Identifying the mutations in a timely fashion is of paramount importance to managing the reproductive options and, if necessary, choosing a preimplantation genetic diagnosis program: the aim is to identify the specific clinical pictures in which a targeted genetic test could guide a personalized diagnostic–therapeutic treatment approach.

### Molecular approaches in the identification of genetic diseases that are transmissible to offspring

It is well known that in 20–25% of cases, perinatal mortality is caused by inherited chromosomal or genetic alterations [127]. Thanks to medical awareness in recent decades, preconception carrier screening has become widely requested. The identification of couples at risk of transmitting a specific inherited disorder to their offspring offers the possibility of making informed reproductive choices to future parents. If the reproductive partner happens to carry a gene alteration for one of the genetic conditions, the pregnancy would be at risk for a child with that disease.

The American College of Obstetricians and Gynecologists has issued standard recommendations for ethnic and general population genetic screening in couples based on reproductive age [128]. Testing is available for more than 2000 genetic disorders, including common diseases, such as sickle-cell anemia, cystic fibrosis, and spinal muscular atrophy, or more complex conditions, such as mental retardation and congenital heart disease.

In this context, genetic counselling is crucial for recognizing the genetic risk, referring patients appropriately and informing patients about genetic issues that are relevant to decision-making [129]. In fact, preconception carrier screening provides genetic information for multiple disorders; thus, all carrier couples can make reproductive decisions based on their results. The tailored genetic test is a crucial tool to improve short-term and long-term outcomes for mothers and their babies [130, 131].

Currently, during the antenatal period, a variety of techniques are available to identify a transmissible disorder to the offspring in the presence of carrier or affected couples. Each of these techniques can be applied only during a specific time period of pregnancy or at different embryo stages in the IVF protocol.

Invasive PND is usually performed on DNA extracted from fetal cells obtained by chorionic villus sampling (CVS) (between the 11th and 13th weeks of gestation) or from amniocytes (from the 15th to the 20th week), and the result is obtained in 7 or 15 days, respectively [132]. The molecular diagnosis for monogenic disease, as we detailed in a previous publication, is carried out by direct mutation analysis when the parental mutations are known or by linkage analysis when the parental mutations are unknown [5, 132]. Paternity verification and contamination analysis are always performed in addition to the specific analytic phases [5].

An increasing amount of interest has been shown regarding the noninvasive prenatal diagnosis (NIPD) of monogenic disease that is able to detect fetal genetic alterations in maternal blood at an early gestational age (approximately 10 weeks). However, although noninvasive prenatal testing (NIPT) of cell-free fetal DNA (cffDNA) for the screening of chromosomes 21, 18, 13, X and Y has been clinically adopted, NIPD remains a challenge [133]. Very recently, NIPD for clinical use has been adopted in cases of sex-linked disorders and RHD [134]. Several studies have tested the application of NIPD in monogenic diseases, such as β-thalassemia, congenital adrenal hyperplasia, and Duchenne and Becker muscular dystrophy [135–137]. The disruptive technology of NGS together with the haplotyping strategy is driving the possibility of using NIPD in clinical cases.

PGT has the same diagnostic motivation as the traditional PND, with the advantage of advancing the timing of diagnosis at the embryo stage. Only disease-free embryos are transferred to the mother, avoiding recourse to therapeutic abortion. Even for couples who are able to conceive naturally, PGT requires the application of IVF techniques, including (a) the collection of gametes from both partners; (b) the fertilization of the oocyte by intracytoplasmic sperm injection (ICSI); (c) the embryo biopsy, which allows one or more cells from the blastomere or trophectoderm to be taken 3 or 5 days, respectively, postfertilization; (d) molecular analysis and (e) the embryo transfer.

PGT protocols are set to start from a small amount of biological sample, ranging from 1 to 10 cells from the embryo at the cleavage stage or blastocyst stage. Conflicting opinions are reported on the detrimental effects of embryo biopsy and mosaicism events between cleavage-stage and blastocyst embryos. Linan et al. demonstrated that the concordance of diagnosis in embryos that were double biopsied on D3 and D5 is 67.8% lower than previously reported, supporting the use of blastocyst biopsies instead of cleavage-stage embryo biopsies [138]. Recently, data from the Preimplantation Genetic Diagnosis International Society (PGDIS) in 2018 showed no difference in the detrimental effects between the embryo stages if experienced operators performed the biopsies [139, 140].

PGT includes whole genome amplification (WGA) to obtain a sufficient quantity of genomic DNA for one or more molecular investigations [141, 142]. Several types of WGA can be used depending on the downstream application [142]. The most used technique for PGT is still represented by “multiplex polymerase chain reaction” (PCR) and capillary electrophoresis analysis for the direct identification of the causative mutation of the disease and the analysis of at least two informative polymorphic markers [the most used are the “short tandem repeats” (STR), microsatellites characterized by short tandem nucleotide repeats] or the analysis of at least 3 polymorphic markers in the event that the causative mutation is not known [5, 143]. However, since PGT tailored to a disease is a laborious and expensive procedure, which is time-consuming in the preliminary phase for the study of the family, several laboratories use genome-wide approaches to analyze gene markers throughout the genome. Alan Handyside tested “karyomapping” based on a single nucleotide polymorphism (SNP) array able to determine the genotype of an individual by analyzing thousands of SNPs distributed throughout the genome [144]. The “karyomapping” involves a “linkage” analysis: with a comparison of the SNPs associated with the causative mutation of the disease to be investigated, that are present in the index case and that are therefore in the parents’ chromosomes, to the SNPs present in the embryo cells, it is possible to identify the presence or absence of the mutation carrier [144–146]. In addition, the density of the SNPs allows a higher resolution in the case of crossings between chromosomes close to the mutated gene. Finally, it is possible to use “karyomapping” in families with combinations of more monogenic alterations or that require HLA compatibility, truly demanding investigations to be carried out with conventional methods. “Karyomapping”, however, loses its effectiveness when it is not possible to establish which parental allele is linked to the genetic alteration; for quantitative analysis of mitotic abnormalities (mosaicisms), “karyomapping” does not directly analyze the mutation and cannot detect de novo mutations. The aim of most new approaches is to use a targeted SNP analysis to detect single mutations or groups of common mutations combined with quantitative haplotype analysis or chromosome count. Recently, a genome-wide protocol using NGS has been tested for the identification of family mutations together with cytogenetic screening in embryo biopsies [147–149]. The protocol is based on an enlarged panel of disease-associated genes (approximately 5000 genes) and enables, in a single workflow, (a) the direct detection of family mutations and the indirect detection through linkage analysis of heterozygous SNPs (PGT-M); (b) a chromosomal translocation (PGT-SR) analysis; and (c) testing for aneuploidies. However, the limitations of a single NGS protocol are related to its inability to detect haploidies, polyploidies, and mosaicisms. In addition, the analysis of consanguineous families is not recommended. Finally, other limitations regarding the limit of detection or the size of the translocation supported by the protocol could be overcome using haplotyping in the presence of the index case.

### Molecular approaches for the optimization of art techniques

Human embryos that are developed in vitro show a great deal of acquired chromosomal abnormalities; for this reason, PGT for aneuploidy (PGT-A) has been developed to select euploid embryos that are suitable for transfer [150].

PGT-A is primarily indicated for couples with advanced maternal age, recurrent implantation failure, recurrent abortions, or severe male infertility. Meiotic errors are one of the main causes of the low success rate (~ 30%) of in vitro fertilization techniques. Randomized studies and meta-analyses have shown that the PGT-A technique does not increase the live birth rate but decreases the miscarriage rate and increases the efficiency of IVF techniques [123, 151].

The evolution of PGT-A techniques started with a limited number of chromosomes analyzed by fluorescence in situ hybridization (FISH) in 1995 [152]. It was soon overcome by the analysis of the whole chromosome set by using different genetic platforms, such as metaphase Comparative Genomic Hybridization (mCGH), array-based Comparative Genomic Hybridization (aCGH), single nucleotide polymorphism (SNP) microarray, quantitative polymerase chain reaction (qPCR), and, most recently, NGS.

Currently, the most commonly used technique is NGS. This method involves the amplification of the genome from a single cell by WGA, the preparation of a DNA library, starting directly from the amplified DNA, and the subsequent sequencing of a pool of libraries in parallel, each identified by a specific “barcode” sequence. Finally, an analysis software that compares the sequences obtained in each sample with respect to the “human hap-map reference genome” allows the identification of the possible presence of chromosome aneuploidies [153]. Literature data confirm that NGS can be successfully applied to the diagnosis of a variety of genetic abnormalities, even in single cells isolated from human embryos following WGA, and has numerous advantages over the technologies traditionally used for PGT-A [153–156].

However, it was soon clear that the gold standard was to develop a method for the analysis of both monogenic diseases and PGT-A at the same time. Indeed, as previously discussed, the very recent innovation for this purpose is the use of NGS to analyze single gene mutations and chromosomal copy number variations to select euploid disease-free embryos [157]. Currently, only a novel mutation continues to be a challenge [140].

## Discussion

Healthy reproduction can be affected by unhealthy environmental and lifestyle factors, increasing paternal and/or maternal age, anatomical or genetic anomalies, systemic or neurological diseases, infections, trauma, and antibody development [158, 159]. As a consequence, infertility can be the result of nongenetic and genetic factors, and it is often multifactorial, polygenic or a combination of both. Presumably, hundreds of genes must interact in a precise manner during sex determination, gametogenesis, complex hormone actions/interactions, embryo implantation, and early development to generate healthy offspring. Indeed, known genetic causes of infertility include chromosomal aberrations, single gene variants and phenotypes with multifactorial inheritance. To date, specific genes and mutations have been confirmed to be associated with infertility phenotypes in males, females or both, and our knowledge regarding the molecular basis of infertility is continually growing. Confirmation of a clinical diagnosis through genetic testing may lead to personalized medical management. Similar clinical symptoms may be the result of different genetic variations. Specifically, in more rare clinical situations, genetic evaluations (counseling and testing) can contribute to the specific identification of the disease or to the confirmation of a suspected diagnosis. The combination of the detailed clinical information provided and the identified genetic cause will allow the development of a personalized diagnostic–therapeutic strategy.

**Fig. 2:**
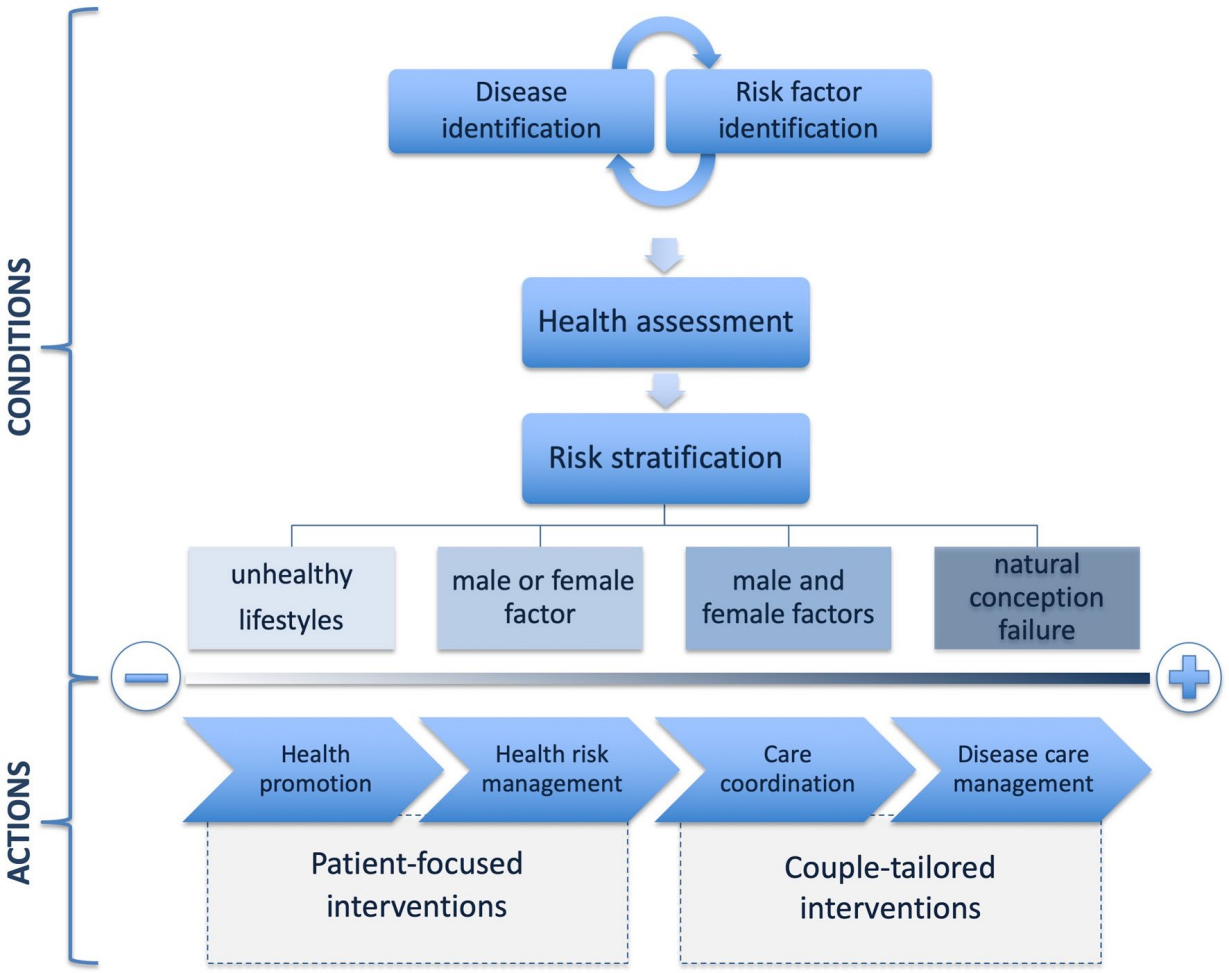
Stratifying the population, through the identification of risk factors and diseases that may be present, allows the organization of targeted diagnostic–therapeutic approaches. The couples in which the reproductive risk is lower are those in which an unhealthy lifestyle was evident in the absence of pathological conditions; in this case, it is necessary to take action based on this information to promote a healthy lifestyle. The reproductive risk increases in couples in which, during the diagnostic phase, the presence of a disease in only one of the partners is identified. In both cases, there are specific interventions aimed at the patient. However, targeted interventions are required in couples with a high reproductive risk, i.e., when both partners are affected by a pathology and after the failure of all methods to achieve pregnancy naturally

The first stage in assessing a couple with potential fertility problems involves the substantial synergy between the patient’s medical history, physical examination, instrumentation analysis (pelvic ultrasound in women) and laboratory tests (sperm analysis in males) to identify any underlying pathology and possible risk factors. Although this evaluation should be performed simultaneously for both partners, the male partner analysis is required only in 18% of cases [160]. Figure 2 shows how the integration of couples’ information can stratify patients on the basis of different morpho-functional aspects and outline already differential diagnostic–therapeutic approaches. When a complete and simultaneous assessment of the couple is not performed, the fertility prognosis is compromised, and the opportunity to improve the health outcomes of each subject is lost. For this reason, it is essential to accurately collect, for each member of the couple, data regarding the personal history (life habits, smoking, alcohol use, drug use, sports activities, etc.) and the medical history (genital characteristics at birth and in the first years of life, pubertal development, and any past disease affecting the genital system). The medical history evaluation must focus on the presence of possible metabolic, endocrine, and genetic disorders with the aim of highlighting elements that can lead to subsequent, specific diagnostic tests. At this stage, it is important to identify patients who can benefit from predictive genetic testing: the use of targeted multigene panels can become a useful tool that, by allowing the identification of further causes of infertility, may improve genetic and reproductive counselling and patient stratification. In fact, some cases of infertility are due to unknown genetic mutations; for this reason, collecting data is mandatory to increase the detection rate (which is currently 80%) and to reduce the percentage of idiopathic infertility.

Many couples learn that they are at high risk of having a child that is affected by a genetic disease only when the woman is already pregnant or after giving birth. Thus, a detailed family history for a couple planning to undergo ART is a useful tool for identifying genetically at-risk couples and can improve the medical care of these patients. A number of important factors must be considered when collecting a detailed family history in the context of family planning [129]. These include the history of the patient’s pregnancy (e.g., multiple abortions may indicate a chromosomal abnormality), the degree of kinship (particular attention should be given to first- and second-degree relatives who may have a history of mental retardation, learning difficulties, progressive muscle weakness, early cataracts, infertility, motionless birth, recurrent miscarriages, and coagulation disorders), the consanguinity of the couple [129], and the ethnicity of both groups of grandparents. Furthermore, references to other specialists (e.g., fetal maternal medicine, reproductive endocrinology, gynecology) should be considered to exclude maternal/paternal infertility causes. In the case of identification of pathologies, in one or both partners, standard diagnostic algorithms will be used, while in specific situations, integration will need to be made with specific diagnostic procedures that require integration with eligibility criteria to access ART.

NGS-based strategies offer the opportunity not only to optimize genetic testing in reproductive medicine (since in one step, it may be possible to analyze the potential causes of infertility, perform carrier screening, and support ART) but also to tailor the therapeutic decision on the basis of the specific genomic features of the patients. Common observation reveals that patients with the same diagnosis may respond differently to therapies, and it is becoming increasingly evident that this differential responsiveness may be due to specific DNA variations at the genomic level. The challenge for future medicine is to move from a population-based view to an individually based one. Novel technologies are driving this revolution. In the context of reproductive medicine, for example, it has been established that specific single nucleotide variants in the genes encoding for hormonal receptors are able to influence the efficacy of hormonal therapies in inducing ovulation [161]. In addition, other molecular features of the patients should be taken into account before starting therapeutic protocols to avoid potential side effects. An opportunity comes from the example of the BRCA test. Indeed, *BRCA1* and *BRCA2* are the most common genes related to hereditary breast and ovarian cancers. Molecular screening for these genes has greatly increased in recent years due to technological simplification and the availability of specific drugs that are suitable for patients with mutations in these genes. As a consequence, several affected and nonaffected young females have been found to be carriers of a BRCA mutation. This information should be taken into account when reproductive choices are planned, considering the potential risk of treating these patients with hormonal therapy.

## Conclusions

Currently, both the entire diagnostic pathway and the effectiveness of genetic analysis for infertility suffer from an approach that is ineffective: only a few genetic variables are studied, each through a specific molecular diagnostic procedure. This makes the process of genetic investigation fragmented and cumbersome, with a negative impact on the couple, in addition to the psychological distress caused by infertility. Therefore, despite the large potential of genetic tests to provide real insights into infertility causes, this crucial tool is still used without a method tailored to diagnostic needs regarding infertility. However, recent developments in new sequencing technologies have made it possible to compact one or more tests into a single NGS-based analysis, thus reducing diagnostic costs and time. The European Society of Human Genetics (ESHG) and the European Society of Human Reproduction and Embryology (ESHRE) have recently issued a recommendation for the development and introduction of extended carrier screening [162]. Although it is difficult to predict at this time how much the diagnostic yield of genetic tests for the different subtypes of male and female infertility will increase, it is realistic to expect a decrease in the current percentage of idiopathic infertility.

The general state of health in the reproductive environment is gaining increasing attention and clinical relevance. Therefore, reproductive specialists have the task of evaluating infertile couples by considering both their general and reproductive health, since the relative conditions of comorbidity can influence their reproduction. Medicine is undergoing an important transformation from a reactive to a preventive approach: the future will focus on the integrated diagnosis, treatment and prevention of diseases in individual patients.

## Data Availability

Not applicable.

## Abbreviations

aCGH: array-based Comparative Genomic Hybridization
AOA: assisted oocyte activation
ART: assisted reproductive technology
AZF: azoospermia factor
cffDNA: cell-free fetal DNA
CFTR: Cystic Fibrosis Transmembrane Conductance Regulator
CVS: chronic villus sampling
DAZ: Deleted in Azoospermia
DNA: deoxyribonucleic acid
ESHG: European Society of Human Genetics
ESHRE: European Society of Human Reproduction and Embryology
FISH: fluorescence in situ hybridization
FMR: Fragile X Mental Retardation
HLA: Human Leukocyte Antigen
ICSI: Intracytoplasmic Sperm Injection
IVF: in vitro fertilization
mCGH: metaphase Comparative Genomic Hybridization
NGS: next generation sequencing
NIPT: non-invasive prenatal testing
OMIM: Online Mendelian Inheritance in Man
PCR: polymerase chain reaction
PGD: preimplantation genetic diagnosis
PGDIS: Preimplantation Genetic Diagnosis International Society
PGT-A: Preimplantation Genetic Test for Aneuploidy
PGT: Preimplantation Genetic Testing
PND: prenatal diagnosis
POI: primary ovarian insufficiency
qPCR: quantitative polymerase chain reaction
SNP: single nucleotide polymorphism
STR: short tandem repeat
STS: sequence tagged site
WGA: whole genome amplification
